# [Retracted] RNA interference against TRIM29 inhibits migration and invasion of colorectal cancer cells

**DOI:** 10.3892/or.2025.8908

**Published:** 2025-05-05

**Authors:** Weihong Xu, Bin Xu, Yiting Yao, Xiaoling Yu, Hua Cao, Jun Zhang, Jie Liu, Huiming Sheng

Oncol Rep 36: 1411–1418, 2016; DOI: 10.3892/or.2016.4941

Following the publication of this paper, it was drawn to the Editor's attention by a concerned reader that certain of the western blotting data featured in Figs. 4C on p. 1415 and 5D on p. 1416 were strikingly similar to data that had already appeared in other articles written by different authors at different research institutes, one of which has been retracted. Owing to the fact that the abovementioned data had already been published prior to the receipt of this article at *Oncology Reports*, the Editor has decided that this paper should be retracted from the Journal. The authors were asked for an explanation to account for these concerns, but the Editorial Office did not receive a reply. The Editor apologizes to the readership for any inconvenience caused.

